# Systematic reviewers' perspectives on sharing review data, analytic code, and other materials: A survey

**DOI:** 10.1002/cesm.12008

**Published:** 2023-04-10

**Authors:** Phi‐Yen Nguyen, Joanne E. McKenzie, Daniel G. Hamilton, David Moher, Peter Tugwell, Fiona M. Fidler, Neal R. Haddaway, Julian P. T. Higgins, Raju Kanukula, Sathya Karunananthan, Lara J. Maxwell, Steve McDonald, Shinichi Nakagawa, David Nunan, Vivian A. Welch, Matthew J. Page

**Affiliations:** ^1^ Methods in Evidence Synthesis Unit, School of Public Health and Preventive Medicine Monash University Melbourne Victoria Australia; ^2^ MetaMelb Research Group, School of BioSciences University of Melbourne Melbourne Victoria Australia; ^3^ Melbourne Medical School, Faculty of Medicine, Dentistry & Health Sciences The University of Melbourne Melbourne Victoria Australia; ^4^ Centre for Journalology, Clinical Epidemiology Program Ottawa Hospital Research Institute Ottawa Ontario Canada; ^5^ School of Epidemiology and Public Health, Faculty of Medicine University of Ottawa Ottawa Ontario Canada; ^6^ Bruyère Research Institute Ottawa Ontario Canada; ^7^ Department of Medicine, Faculty of Medicine University of Ottawa Ottawa Ontario Canada; ^8^ School of Historical and Philosophical Studies University of Melbourne Melbourne Victoria Australia; ^9^ Leibniz‐Centre for Agricultural Landscape Research (ZALF) Müncheberg Germany; ^10^ African Centre for Evidence University of Johannesburg Johannesburg South Africa; ^11^ Population Health Sciences, Bristol Medical School University of Bristol Bristol UK; ^12^ Interdisciplinary School of Health Sciences University of Ottawa Ottawa Ontario Canada; ^13^ Faculty of Medicine University of Ottawa Ottawa Ontario Canada; ^14^ Evolution & Ecology Research Centre and School of Biological, Earth and Environmental Sciences University of New South Wales Sydney New South Wales Australia; ^15^ Nuffield Department of Primary Care Health Sciences, Centre for Evidence‐Based Medicine Oxford University Oxford UK

## Abstract

**Background:**

There are many benefits of sharing data, analytic code, and other materials, yet these items are infrequently shared among systematic reviews (SRs). It is unclear which factors influence authors' decisions to share data, code, or materials when publishing their SRs. Therefore, we aimed to explore systematic reviewers' perspectives on the importance of sharing review materials and factors that might influence such practices.

**Methods:**

We searched PubMed for SRs published from January to April 2021, from which we randomly allocated 50% to this survey and 50% to another survey on the replication of SRs. We sent an electronic survey to authors of these SRs (*n* = 4671) using Qualtrics. Quantitative responses were summarized using frequency analysis. Free‐text answers were coded using an inductive approach.

**Results:**

The response rate was 9% (*n* = 417). Most participants supported routine sharing of search strategies (84%) but fewer for analytic code (43%) or files documenting data preparation (38%). Most participants agreed that normative practices within the discipline were an important facilitator (78%). Major perceived barriers were lack of time (62%) and suitable sharing platforms (31%). Few participants were required by funders (19%) or institutions (17%) to share data, and only 12% of participants reported receiving training on data sharing. Commonly perceived consequences of data sharing were lost opportunities for future publications (50%), misuse of data (48%), and issues with intellectual property (40%). In their most recent reviews, participants who did not share data cited the lack of journal requirements (56%) or noted the review did not include any statistical analysis that required sharing (29%).

**Conclusion:**

Certain types of review materials were considered unnecessary for sharing, despite their importance to the review's transparency and reproducibility. Structural barriers and concerns about negative consequences hinder data sharing among systematic reviewers. Normalization and institutional incentives are essential to promote data‐sharing practices in evidence‐synthesis research.

## 1 INTRODUCTION

There are several benefits of sharing data, analytic code, and other materials from systematic reviews (hereafter referred to as “review materials”). For readers, gaining access to data and code can provide insight about the review's design and analytic methods, from which they can judge its transparency, strengths and limitations, and risk of biases. Furthermore, access to the underlying data and the analytic code facilitates attempts to verify the review's findings. Publicly available review data can be reused in review updates—for example, as new evidence or new statistical methods become available—or in secondary analyses (e.g., exploring effects of prognostic factors or health equity indicators [1]), without wasting time recollecting the data or contacting trialists for data [2]. For the authors of the review, sharing data alongside their publication is a sign of rigor and transparency, which can improve the credibility of the research to journals and readers [3], and can result in more citations, media exposure, and professional opportunities [4].

In the past decades, the research community has become more supportive of data sharing. Mandates from major public funders, such as one recently issued by the US National Institutes of Health (NIH) [5], are expected to accelerate the normalization of data sharing practices. However, as these initiatives tend to target clinical trials and basic science [6, 7], the evidence synthesis research landscape is still plagued by low availability of data and code [8, 9, 10].

Several factors can discourage authors from sharing data or code when publishing their reviews. Reviewers can be deterred by fear of criticism, of others misusing their data, and of losing potential publications to other researchers who make use of their shared data [11, 12]. Additionally, some researchers might lack knowledge about best practices and available venues for data sharing. At the policy level, support is lacking from institutions, especially universities and journal editors in terms of legal arrangements, official guidelines, institutional and journal policies to regulate, protect and reward authors who share data [3, 13, 14, 15, 16]. Systematic reviewers also face unique challenges not commonly encountered by investigators of other types of research. There still exists a misconception that all data generated from systematic reviews and meta‐analyses is secondary data and easily retrievable from primary studies. Authors of systematic reviews often state (in data availability statements) that review data will be shared upon request, or imply that data sharing is unnecessary because their review did not generate new data [17]. Many types of data files required for replicating or validating a review are incompatible with traditional journal formatting requirements due to their lengths (e.g., search strategies for all databases, list of all excluded studies).

Several studies have explored beliefs and perceptions of authors on data sharing [15, 18, 19, 20, 21, 22, 23, 24]. However, none of these studies has focused on systematic reviewers, who may face these unique challenges or have different perspectives from authors of other study designs. Therefore, we aimed to explore the perspectives of systematic reviewers on the importance of sharing review data, code and other materials, and factors that might influence such practices.

## 2 METHODS

This study was conducted as part of the REPRISE (REProducibility and Replicability In Syntheses of Evidence) project. The REPRISE project consists of a suite of four studies that aim to investigate various aspects of transparency, reproducibility, and replicability of systematic reviews of the effects of health, social, behavioral, and educational interventions [25]. Methods for all studies in the REPRISE project were prespecified in the published protocol [25]; all deviations are detailed in Supporting Information: File S1. In the present paper, we report the results of Study 2b. In a companion paper, we report the results of Study 2a, where we explored systematic reviewers' views on replication of systematic reviews.

### 2.1 Ethical approval and informed consent

This study was approved by Monash University Human Research Ethics Committee (institutional review board protocol no. 27270). All participants provided their informed consent to taking part in the survey at the start of the survey questionnaire. All responses were deidentified and anonymised using unique identification numbers.

### 2.2 Creation of the sampling frame

We systematically searched PubMed for systematic reviews indexed from January 1st to April 30th, 2021, using a pragmatic search strategy developed by an information specialist (SM)]: (meta‐analysis[PT] OR meta‐analysis[TI] OR systematic[sb]) AND 2021/01/01:2021/04/30[EDAT], with no language restriction. We selected this date range as it occurred relatively close to the time when we started setting up the survey. Given the survey required systematic reviewers to recall whether they shared any materials of their published systematic review, we wanted to keep the length of time between review publication and survey completion at a minimum. Records were imported to Endnote X9.3.3 for removal of duplicates. We then sought the corresponding authors' name, email address, and affiliation using two methods of parsing data (implemented in R v.2.13). Specifically, parsing public information from PubMed online articles using the *easyPubMed* package [26], and parsing text from PDF full‐text files using the *pdftools* package [27]. If multiple corresponding authors were listed, only the first corresponding author's details were recorded. The collected information was reviewed for accuracy by one author (Phi‐Yen Nguyen or Matthew J. Page). We then excluded reviews which had been included in REPRISE Study 1 [28], or reviews coauthored by any of the investigators of this study. The reason for the former exclusion criterion was to reduce author burden, since these authors will be contacted as part of another REPRISE study. If two or more reviews had the same corresponding author, we only included the most recent review. We screened all titles for correction, corrigendum, erratum, author's reply, or response related to a systematic review, and only included the systematic review in the sampling frame.

The sampling frame was initially stratified into countries in which the corresponding author was based. The order of entries within the sampling frame was then randomized using the function RAND() in Excel. Within each country stratum, we assigned the first 50% of the randomized entries into the companion survey on replication of systematic reviews (Study 2a) and the other 50% into this survey (Study 2b).

### 2.3 Survey sampling and distribution

There were two phases of survey distribution. In Phase 1, we administered the survey to a stratified random sample of 300 authors. We stratified the sample by country (based on the corresponding author's first affiliation) and used simple random sampling to select the reviews within each stratum (country). The number of reviews selected per country was proportional to the number of reviews in that country (proportional allocation) [29]. All random selection was performed using the RAND() function in Excel. In Phase 2, we administered the survey to all remaining participants in the sample.

We took a two‐phased approach for a number of reasons. First, a random sample of 300 participants with a high response rate would provide sufficient precision and generalizable results to the population of systematic reviewers. However, we anticipated that the response rate might be low based on similar surveys (e.g., 15%), necessitating the need to increase our sample size to obtain a greater range of viewpoints (recognizing the diminishing generalizability). Second, we wanted to determine how many emails were unsuccessfully delivered, and finally, how effective our schedule of reminders was for attaining responses.

Both phases of the survey were delivered online using the survey platform Qualtrics [30]. Phase 1 commenced on July 27th, 2021, whereby an invitation was sent to the 300 randomly selected authors (Supporting Information: File S2). This invitation was followed by up to three reminders, sent at 3‐week intervals, in the case of nonresponse. Given the low response rate observed in Phase 1, we commenced Phase 2 on October 14th, 2021, with all remaining authors sent an invitation and up to three reminders at 2‐week intervals in the case of nonresponse. We shortened the period between reminders to 2 weeks in Phase 2 because very few responses were recorded beyond the first few days following reminder emails in Phase 1. In both phases, we dealt with notifications of unsuccessful delivery by searching the institutional website of the corresponding author to seek an accurate email address; if no such email address could be found, we used the next available corresponding author email address, when available. Phase 2 of the survey distribution was closed on December 10th, 2021.

### 2.4 Survey content

Before launching the survey in Phase 1, we sent a preliminary version of the survey to five experienced systematic reviewers known to the corresponding author, all of whom reviewed the preliminary version and provided feedback on the wording and content, which we incorporated. The final version of the survey comprised two sections (Supporting Information: File S3). The first section surveyed the participants' opinions on the frequency that different types of review materials should be shared, and the perceived factors that might facilitate or disincentivise sharing. The participants were also asked about their personal practice of data sharing, and reasons for sharing or not sharing data in their own systematic reviews. Items in this section of our survey were informed by previous surveys on researchers' views on sharing of data, code, and other materials for other study designs [19, 24, 31, 32, 33, 34, 35, 36]. The second section surveyed the demographic and professional background of the participants, including country of residence, discipline of research, whether they identified as methodologists, statisticians, early‐career researchers or PhD students, and their experience with conducting systematic reviews. The survey consisted of binary (yes/no) and 7‐point Likert‐scales, and open‐ended questions to allow the gathering of free‐text comments.

### 2.5 Data analysis

We used descriptive statistics (frequency and percentages) to summarize the responses of all survey questions. For ease of reporting percentages in the text, we only report the cumulative percentage of positive response options for 7‐point Likert scale questions (i.e., sum of “somewhat agree,” “agree,” and “strongly agree”) for each item. Percentages of neutral and negative responses are also available in the corresponding figure and table. Percentages were calculated based on the available data for each item; we did not impute missing data. All analyses and graphs were generated in R v.4.1.2.

We used an inductive approach [37] to coding of free text comments. One author (Phi‐Yen Nguyen) read each free text response and either (i) coded them using an existing code, generated from existing response options in the survey or from coding a previous respondent's questionnaire or (ii) assigned a new code which captured the meaning and content of the text. For example, for the question on reasons for practising data sharing, “to follow with the PRISMA reporting recommendations” was recoded into the existing option “to conform to expectations within my discipline.” Other free‐text answers such as “To provide an educational framework for future learners” and “To show to editors/reviewers that we used good practices” were assigned a new code, “To demonstrate good practices.” As each subsequent free text response was read, existing codes were reviewed and revised, and new codes were added, when necessary. All codes assigned were reviewed by another author (Matthew J. Page) and any discrepancies were resolved via discussion. We present the frequency and percentage of each code and provide illustrative quotes for each.

## 3 RESULTS

### 3.1 Overview

**Figure 1:**
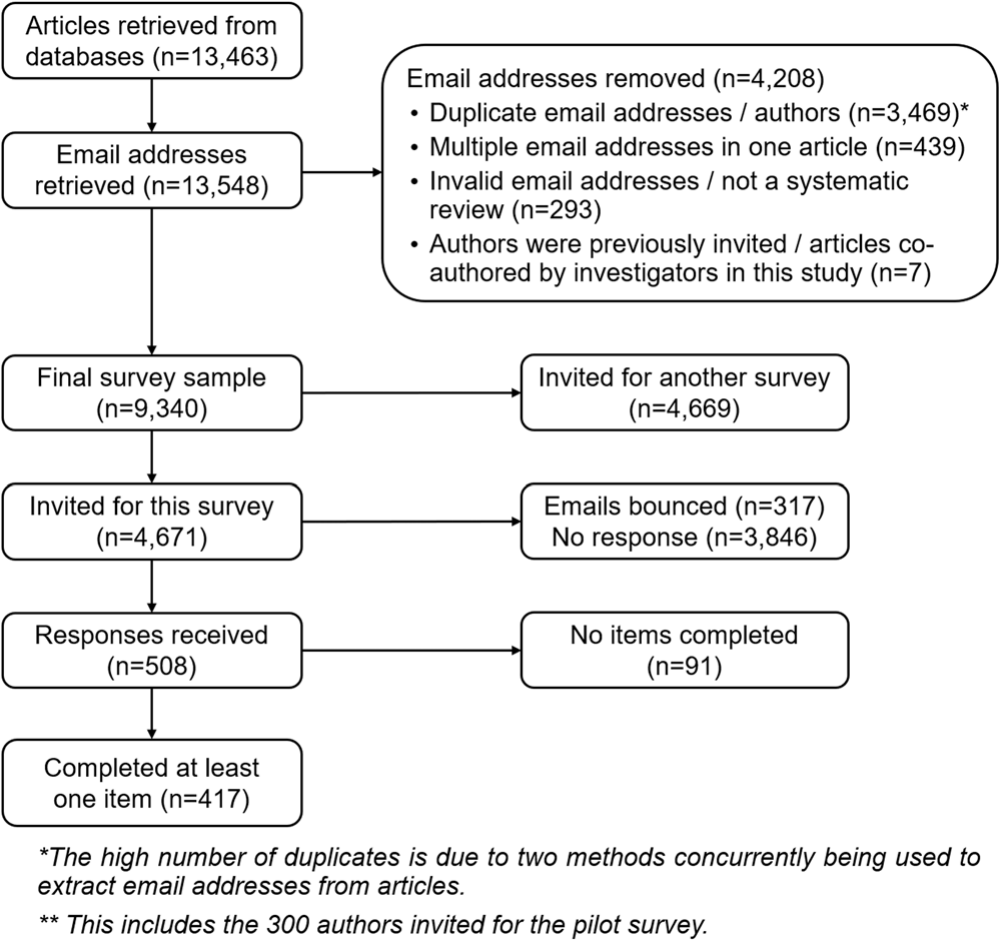
Flowchart of recruitment and data collection.

As reported in the companion survey (Study 2a), we retrieved 13,463 records from the PubMed search, from which we obtained 13,548 email addresses. After removing 4208 email addresses for various reasons (mainly because they were duplicates; Figure 1), our final sampling frame consisted of 9340 unique email addresses, of which we randomly selected 4671 (50%) for this survey. Across both phases of survey distribution, among the emails sent out, 317 (7%) returned with notifications of failed delivery. Of the 4354 successfully delivered emails, 508 participants consented (12%), with 417 completing at least one survey item (9%).

### 3.2 Participant characteristics

Participants were based in 47 countries. The region where most participants resided in was Europe (42%), followed by the Americas (27%) and Western Pacific (14%), with the top countries being the United States (12%), the United Kingdom (12%), and Australia (9%). The majority were affiliated with a university (84%) or a hospital (29%). The most common disciplines participants worked in were medicine (including dentistry) (52%), allied health (including sports sciences and rehabilitative therapy) (21%), and public health and epidemiology (16%). More than half the participants (52%) identified as a methodologist, while 10% identified as a statistician. Twenty percent of participants were PhD students and 31% had completed their PhD within the last 5 years. Almost all participants (91%) reported having conducted research for at least 3 years. A large percentage (78%) had coauthored at least three systematic reviews throughout their career. Almost all participants (92%) slightly, mostly or completely supported the open science movement (Table 1).

**Table 1:** Participant characteristics.

	Response *n*/*N* (%)
Country of residence	
Europe	122/290 (42%)
Americas	78/290 (27%)
Western Pacific	42/290 (14%)
Eastern Mediterranean	14/290 (5%)
Africa	20/290 (7%)
South‐East Asia	14/290 (5%)
Type of institutions affiliated with^a^	
University	308/368 (84%)
Hospital	108/368 (29%)
Government department	11/368 (3%)
Commercial company	5/368 (1%)
Research institute not affiliated with any of the above	25/368 (7%)
Other institutions	5/368 (1%)
Discipline	
Allied health—not elsewhere specified	49/331 (15%)
Agriculture	3/331 (1%)
Biological sciences, including genomics	9/331 (3%)
Economics	3/331 (1%)
Engineering	3/331 (1%)
Environmental sciences	1/331 (0%)
Epidemiology	11/331 (3%)
Medicine, including dentistry	172/331 (52%)
Nursing	14/331 (4%)
Pharmacy and pharmacology	9/331 (3%)
Psychiatry and psychology	28/331 (8%)
Public health—not elsewhere specified	43/331 (13%)
Rehabilitative allied health and sports sciences	20/331 (6%)
Social sciences and social work	6/331 (2%)
Statistics	3/331 (1%)
Veterinary sciences	4/331 (1%)
Other	7/331 (2%)
Job description	
Methodologist	185/356 (52%)
Statistician	37/357 (10%)
PhD student	72/354 (20%)
Completed PhD within the last 5 years	109/354 (31%)
Years of experience in research	
Less than 3 years	30/357 (8%)
3–10 years	158/357 (44%)
More than 10 years	169/357 (47%)
Number of systematic reviews authored or coauthored	
0	3/357 (1%)
1	39/357 (11%)
2	38/357 (11%)
3–10	171/357 (48%)
More than 10	106/357 (30%)
Opinion on open science	
Completely opposed	0/356 (0%)
Mostly opposed	4/356 (1%)
Slightly opposed	13/356 (4%)
No opinion	10/356 (3%)
Slightly support	35/356 (10%)
Mostly support	149/356 (42%)
Completely support	145/356 (41%)

^a^ Participants could select more than one option and therefore percentages across options can add to greater than 100%.

### 3.3 Participants' experience with data sharing in their most recent systematic review

Seventy percent of participants had registered the protocol of their most recently published systematic review. Furthermore, they reported to share the following review information most commonly (Table 2): the full line‐by‐line search strategy for all databases (77%), template data extraction forms (34%), and files containing data used in all statistical analyses, such as Review Manager (.rm5) or Comprehensive Meta‐Analysis (.cma) files (28%). Analytic code (13%) and files used to prepare data (e.g., data conversion, calculation of missing data) for analysis (13%) were the least commonly shared. Only 11 (3%) participants reported to have shared all eight types of review material specified in the questionnaire (Supporting Information: File S3). Other materials shared included citations of excluded full texts (*n* = 8), detailed risk‐of‐bias assessments (*n* = 8), the review protocol or registration record (*n* = 7), reporting checklists such as a PRISMA checklist (*n* = 4), details of screening and/or data collection processes (*n* = 3), or a sample search strategy from one database (*n* = 3), among others (Supporting Information: File S4). Among those who shared files, 73% indicated that they assigned a digital object identifier (DOI) and 62% assigned a license (e.g., CC‐BY) to their files (Table 2).

**Table 2:** Characteristics of participants' latest published systematic review.

	Response *n*/*N* (%)
Registered the review protocol	244/351 (70%)
Received private requests for data files, analytic code, or other materials associated with review	53/342 (15%)
Items shared alongside most recent systematic review^a^	
Full line‐by‐line search strategy as run in all databases	320/417 (77%)
Template data collection form(s)	140/417 (34%)
File(s) containing data used in all meta‐analyses, subgroup analyses, sensitivity analyses, and other analyses	117/417 (28%)
File(s) containing citations of all records that were screened	116/417 (28%)
File(s) containing (unprocessed) data extracted from included studies	96/417 (23%)
Metadata which describes the contents of the shared file(s) to aid interpretation and reuse	60/417 (14%)
Analytic code used to generate results	55/417 (13%)
File(s) containing conversions to prepare data for analysis	53/417 (13%)
Other materials	47/417 (11%)
Methods of sharing data files or analytic code for most recent systematic review^a^	
Uploaded as a supplementary file on the journal website or as an appendix within the manuscript	129/157 (82%)
Uploaded to a general‐purpose open‐access repository (e.g., Open Science Framework, Zenodo, GitHub, figshare)	36/157 (23%)
Uploaded to an institutional repository	15/157 (10%)
Uploaded to my personal website	6/157 (4%)
Other methods	5/157 (3%)
DOI associated with shared data files or analytic code for the most recent systematic review	
None	29/110 (26%)
All files	61/110 (55%)
Some files	20/110 (18%)
License associated with shared data files or analytic code for most recent systematic review	
None	36/97 (37%)
All files	47/97 (48%)
Some files	14/97 (14%)
Reasons for sharing data files or analytic code for most recent systematic review^a^	
To enhance the transparency of my systematic review	139/157 (89%)
To enhance the reproducibility of my systematic review	123/157 (78%)
The journal required sharing of data files or analytic code	49/157 (31%)
To conform to expectations within my discipline	38/157 (24%)
To gain more citations for my systematic review	22/157 (14%)
To increase my chances of success in future job applications	11/157 (7%)
My institution requires sharing of data files or analytic code	9/157 (6%)
The funder required sharing of data files or analytic code	7/157 (4%)
Other reasons	10/157 (6%)
Reasons for not sharing the file(s) containing data used in all meta‐analyses for most recent systematic review^a^	
The journal did not request sharing of any data file(s)	148/265 (56%)
I did not conduct any meta‐analyses, subgroup analyses, sensitivity analyses, or other analyses	78/265 (29%)
I did not know how or where to share my data file(s)	56/265 (21%)
I was too busy to prepare the data file(s) such that they could be used by someone external to the project	54/265 (20%)
I did not see any value in sharing my data file(s)	52/265 (20%)
I never considered sharing my data file(s)	49/265 (18%)
I was concerned about misuse of the data file(s)	39/265 (15%)
I wanted to protect my intellectual property	37/265 (14%)
My organization does not reward me for sharing data files	35/265 (13%)
I planned to conduct additional analyses of the data collected	35/265 (13%)
My organization has no one to advise on how to share data file(s)	25/265 (9%)
I was concerned that others would criticize the data file(s)	19/265 (7%)
I was contractually obliged to not share the data file(s)	5/265 (2%)
I wanted to protect commercially sensitive information	2/265 (1%)
Other reasons	10/265 (4%)

^a^ Participants could select more than one option and therefore percentages across options can add to greater than 100%.

Most participants (82%) reported that they shared these files as supplementary files on the journal website or as an appendix within the manuscript. Others uploaded these files to a general‐purpose open‐access repository (23%) or other venues (17%) (Table 2). The repositories used by the participants included the Open Science Framework (*n* = 4), figshare (*n* = 4), and Github (*n* = 3).

The main reasons participants provided for sharing their review data and code in their most recent review were to enhance transparency (89%) and reproducibility (78%) of their reviews (Table 2). Other factors were adhering to journal requirements (31%), expectations within the discipline (24%), and expectation of more citations associated with sharing data (14%). Some participants provided further reasons in the free‐text responses (Supporting Information: File S5), stating that sharing data was their way of demonstrating good research practices to editors or peer reviewers (*n* = 2), and that sharing would likely increase their chance of journal acceptance (*n* = 1) and facilitate future updates by others (*n* = 1).

Authors who did not share data or code in their most recent review reported the role of journals in influencing their decision not to do so, with 56% of participants citing the lack of journal requirements as a reason for not sharing their files (Table 2). A third of participants (29%) reported not sharing because the review did not include any statistical analysis (e.g., meta‐analyses and sensitivity analyses). Other participants did not see the value in sharing their data (20%), supported by the finding that only 15% of participants had ever received private requests for data after their most recently published review. Other reasons are a lack of knowledge of how and where to share data files (21%) and lack of time (20%). Potential reasons for not sharing which received a positive response from less than 20% of the participants are shown in Table 2. Some authors stated other reasons for not sharing data in free‐text responses (Supporting Information: File S6). They stated that they were not permitted by the data's owner (*n* = 1) or the funding agency (*n* = 1) to share data, or were otherwise bound by confidentiality requirements (*n* = 2). Others commented that sharing all data was cumbersome for journals and readers (*n* = 1), and also for authors who had to prepare and annotate the data (*n* = 2).

### 3.4 Opinions on the frequency of data sharing

**Figure 2:**
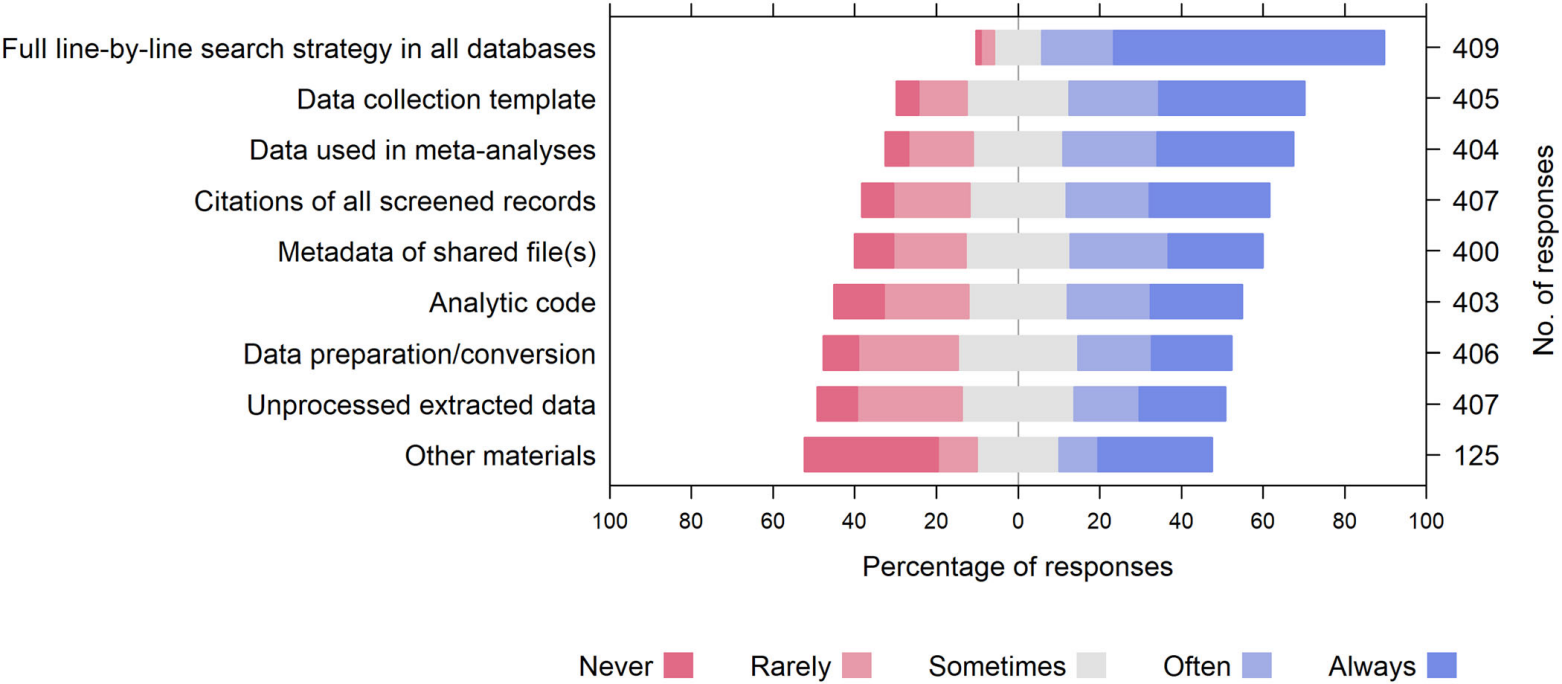
Participants' opinions on how often systematic review files should be shared publicly.

Most participants (84%) agreed that the full line‐by‐line search strategy for all databases should be frequently shared. Other file types that received positive responses (“often” or “always”) from more than 50% of participants were data collection templates (58%), files containing data used in analyses (56%), and citations of all screened records (50%). File types receiving positive responses from less than 50% of the participants, included meta‐data files describing the data (47%), analytic code (43%), files explaining data conversions or preparation (38%), and files containing unprocessed extracted data (37%) (Supporting Information: File S7, Figure 2).

Other file types that participants suggested should always be shared included the protocol or registration record (*n* = 7), details of screening and/or data collection processes (*n* = 5), risk‐of‐bias assessments (*n* = 4), citations of all excluded full texts (*n* = 3), a detailed outline of the review workflow (*n* = 3) and reporting checklists such as PRISMA (*n* = 2), among others (Supporting Information: File S8).

### 3.5 Opinions on factors influencing data sharing

**Figure 3:**
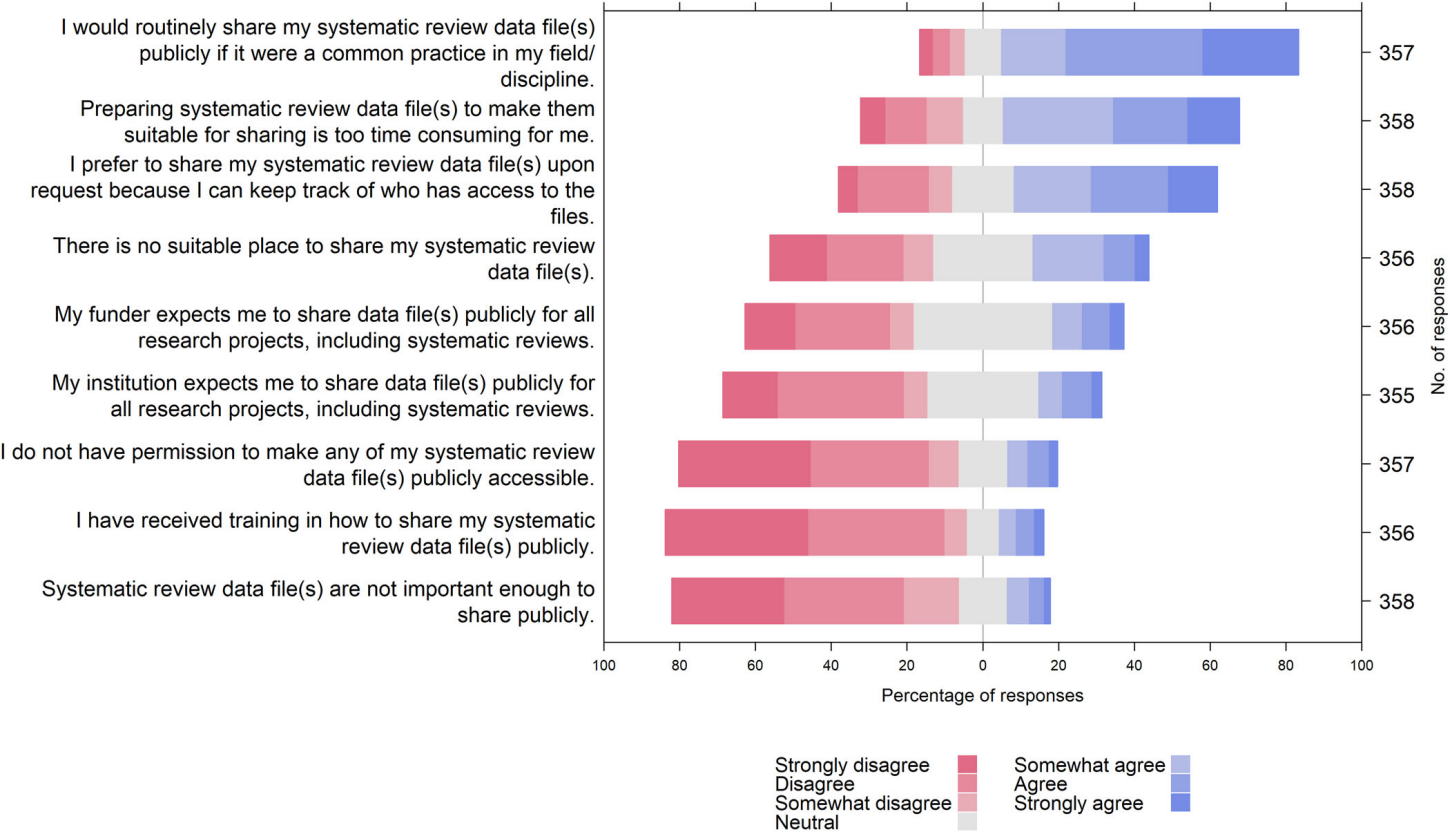
Participants' opinions on factors influencing decisions to share review materials.

Participants were asked about factors that could influence their decision to share systematic review data or analytic code routinely in the future (Supporting Information: File S7, Figure 3). A major driving factor was the normative practices within the discipline, with 78% agreeing that they would share their review data routinely if it was a common practice. Only 19% of participants were required by their funders to share data files publicly, and only 17% by their institutions. In addition, 13% believed they were not authorized to make their data files publicly accessible. Another important factor was the technical barrier, with only 12% of participants having received training in data sharing, 31% unable to find a suitable platform to share data files and 62% finding it too time‐consuming to prepare data for sharing. Others (54%) preferred to share data upon private request, so as to keep track of who has access to the files.

### 3.6 Opinions on potential consequences of data sharing

**Figure 4:**
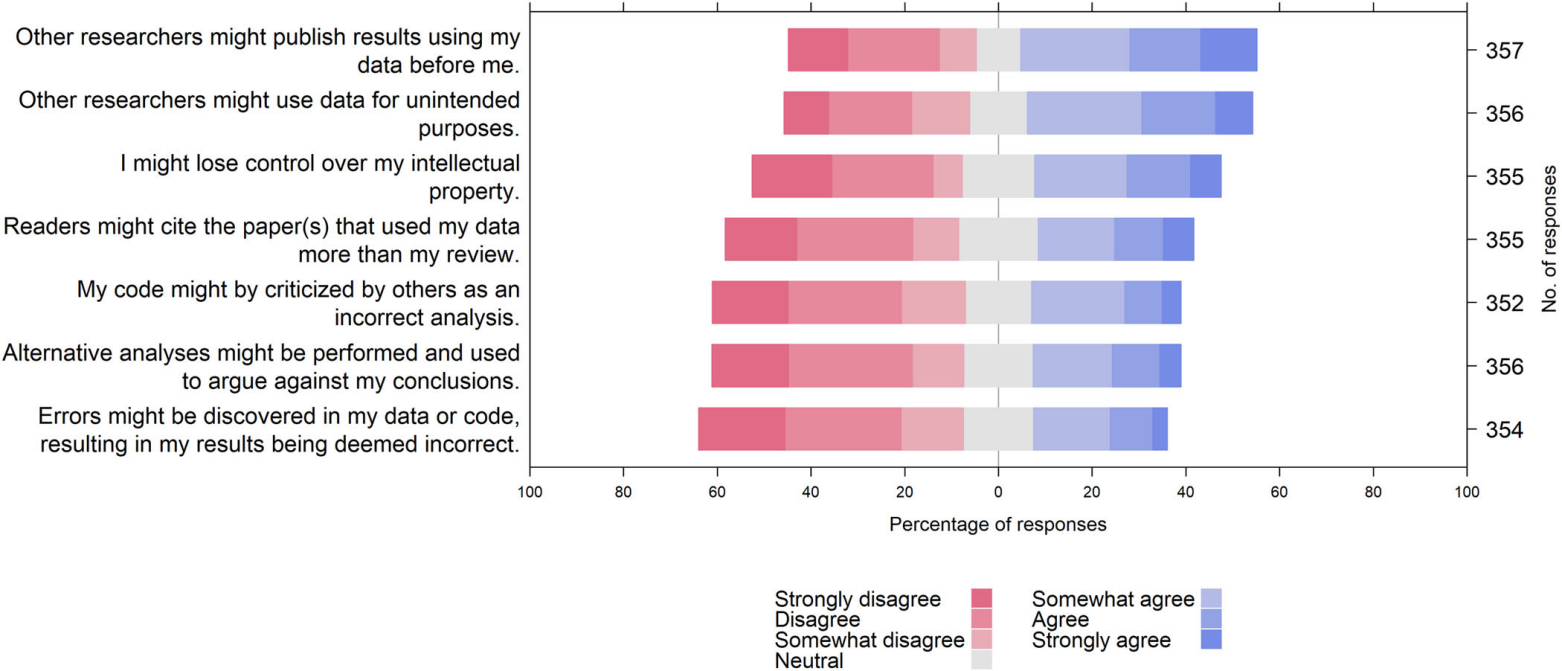
Participants' opinions on potential consequences of sharing review materials.

The most common concerns were that other researchers would publish the results using the shared data (50%) and subsequently they would lose citations to those other reviews (33%). Others worried about misuse of data for unintended purposes (48%) or infringement of intellectual property (40%). A third of participants reported worrying that data sharing might invite alternative analyses that argue against their conclusions (31%), invite criticisms about their incorrect analysis (32%) or identify errors in the data or code (29%) (Supporting Information: File S7, Figure 4).

## 4 DISCUSSION

In this study, we surveyed systematic reviewers on several issues surrounding data sharing in systematic reviews. In summary, many participants reported to be in favor of routine sharing of some materials associated with the review, particularly the search strategies, template data collection form, and data used in analyses. However, there was a disconnect between participants' expressed support and actual sharing practice in their most recent review. In addition, participants expressed concerns about the potential negative consequences of sharing review materials and identified factors that might incentivise data sharing. Despite the low response rate, findings from the survey provide valuable insights among participating systematic review authors in health research.

### 4.1 On the frequency of sharing review materials

The percentages of participants who reported sharing systematic review materials are comparable to data in other studies surveying researchers working on other study designs [15, 22, 33, 38], but higher than the actual rates of data sharing observed in studies [9, 10, 39]. This can perhaps be attributed to a high percentage of methodologists and open science supporters in our sample, as well as social desirability bias [40]. The more frequent sharing of search strategies can likely be attributed to the PRISMA statement [41], which recommended this practice back in 2009; whereas, recommendations for the sharing of other materials were not provided. The inclusion of an item on data availability in PRISMA 2020 [42] might improve sharing rates in future. Other types of review materials that provide details of the data preparation and statistical analyses, including analytic code, files showing data conversion and imputations, and unprocessed extracted data, were not as frequently shared. This infrequent sharing of these review materials has been reported in other studies [10, 39, 43]. It is possible that some participants did not report sharing analytic code simply because their meta‐analytic software do not require coding (e.g., Review Manager or Comprehensive Meta‐Analysis).

### 4.2 On facilitator and barriers of data sharing

Three main themes emerged as strategies to improve data sharing in systematic reviews: building a culture that normalizes data sharing, providing personal incentives to researchers, and establishing infrastructure that facilitates data sharing.

First, a culture that normalizes data sharing is still in its infancy in evidence synthesis research. Participants overwhelmingly agreed that they would practice data sharing if it became standard practice in their discipline. This might be achieved through various strategies implemented at research institutions, such as integrating data sharing into research training curriculum, and monitoring and recognizing data sharing as an academic output at the institutional level [44]. In addition, funding agencies and journals also play critical roles in promoting data sharing. Besides the financial incentives, some funding agencies also have pre‐existing mechanisms to monitor and enforce data sharing among recipients [45]. Studies have shown that open data policies in academic publishing are effective in increasing the frequency of data sharing [16, 46, 47, 48, 49]. In our survey, however, few participants believed they were expected by funding agencies, their institutions, or journal editors to share review materials. Another perceived barrier is the lack of a universal guidance on which data types should be shared and how. Such frameworks exists for clinical trials [50] and genomic studies [51], but ironically not for systematic reviews, which also underpin important policies and clinical guidelines. Valuable lessons can be learned from ecological sciences' successful initiatives, ranging from mandatory public data archiving policies enforced by leading journals [52], to international research networks establishing policies governing the responsibility of data providers and data users [53].

Second, for our participants, the perceived negative consequences of sharing review materials tended to outweigh the perceived societal and personal benefits. Approximately a third of participants expressed concerns about “scooping” and, by extension, loss of academic productivity, potential citations and ownership of research data. Moreover, fear of inviting criticism or conflicting conclusions arising from reanalysis of their data, though raised by fewer participants, are also important considerations. Such concerns might be more pronounced for data that are more prone to subjectivity and inter‐rater differences (e.g., risk of bias judgements, GRADE assessments) [54, 55]. Researchers might be concerned about their data being misused or misinterpreted without their knowledge, such as in commercial exploitation or misleading secondary analyses, causing damage to their credibility [12, 16, 22]. Other surveys echoed these perceptions among researchers conducting other types of studies [13, 14, 22, 24, 31, 32, 35, 36]. Recent evidence suggests that many of these concerns may be unfounded. Data sharing has been shown to enhance perceived credibility of a study [32], invite coauthorship [11, 34], and equally attract citation or funding opportunities [4]. In fact, sharing data through formal channels will provide the authors with the DOI and time‐stamps to establish higher priority of discovery in the future [56], especially when they want to use the same data to update a meta‐analysis. Moreover, universities and research institutions are gradually moving away from using journal‐based metrics for hiring and promoting decisions [57, 58] which should mitigate concerns about publication opportunities in the future.

Given our participants' negative perceptions toward data sharing, it is important that messages about the benefits of data sharing be communicated more widely to researchers. In addition, data sharing practices should be encouraged via rewarding mechanisms [13, 15, 33, 35, 36, 59]. Existing models which acknowledge sharing of data can be adopted, such as open practice badges, data citation statistics, or public attribution to data owners [60, 61, 62]; valuable lessons can be learned from the successes of publicly available data registries [63, 64, 65]. For systematic reviews, credit should similarly be awarded to researchers who share review materials for reuse in future replications or updates of reviews.

Last, the processes and infrastructure required for data curation and management (i.e., skills, tools, and sites for data storage) need improvement. Venues to store and disseminate data were not deemed an issue by many of our participants, thanks to advances in open‐access repositories and data‐sharing platforms [66]. However, most (79%) participants reported that they lacked training in data sharing. They also found the process of curating review materials for public sharing time consuming. Annotation and preparing metadata has long been acknowledged as time‐consuming, or even costly [16, 31, 34]. Systematic reviewers often report not having received dedicated funding to support the conduct of their review [28], and so are less likely to have the budget for preparation of data which other study designs, such as trials and cohort studies, tend to have. This issue is particularly pertinent for individual participant data (IPD) meta‐analyses, in which access to data is often governed by data use agreements between the reviewers and trial investigators [67]. The prospect of lengthy contractual negotiations to obtain permissions for data sharing would likely deter reviewers from sharing meta‐analytic data altogether. Research institutions are in a better position to address challenges related to training and time constraints, for example, by creating a data management department to provide data stewardship services and organize training for researchers [68, 69]. Publicly available tools, such as data management templates [70], are effective venues to introduce new systematic reviewers to efficient data management workflows.

While the open data movement has achieved major accomplishments in clinical trials [71], it has yet to gain momentum in evidence synthesis. Given the extensive use of systematic reviews in policymaking and clinical practice guidelines, it is imperative that similar levels of resources and effort be dedicated to promote data sharing in evidence synthesis. Researchers are strongly motivated by external factors [11], and behavioral models suggest that both institutional factors and individual‐level factors affect data‐sharing behaviors [48]. Our survey confirms that multifaceted approaches, addressing the identified barriers and facilitators, are required to promote data‐sharing behaviors among systematic reviewers.

### 4.3 Limitations and strengths

There are several strengths of our study. The sampling frame was designed to capture systematic reviewers across a diverse range of disciplines and geographic locations. In addition to quantitative questions, we also used open‐ended questions to help further explain the quantitative responses. Two authors were involved in coding of free‐text responses.

Nonetheless, our study was not without its limitations. Although we had adopted several strategies to minimize selective nonresponse, such as avoiding the term “survey” in the email subject heading, trialing the survey distribution, keeping the survey's length to approximately 15 min, and sending reminders to nonrespondents [72], our response rate was low (9%). As a result, we have refrained from reporting confidence intervals, since the low response rate could have compromised the random sampling process and potentially affected the validity of the confidence intervals. Without demographic information of nonresponders, we cannot assess the extent of nonresponse bias in our sample. Since the survey was written in English, it is possible that participants not speaking English as a first language may have been discouraged from responding or may have misinterpreted some of the survey questions [73]. While 12% of reviewers in the sampling frame were based in China, only 0.5% of our survey participants were based in China. Therefore, our sample may not necessarily represent the geographic distribution of systematic reviewers. Moreover, 52% of our responders identified as methodologists, who may have different perspectives on data sharing to systematic reviewers not doing methodological research. Given the cross‐sectional design, our survey only reflects the level of data sharing reported by systematic reviewers in 2021.

## 5 CONCLUSION

Systematic reviewers reported being in favor of routine sharing of some review materials (e.g., search strategies) but not others (e.g., unprocessed data, analytic code). Insufficient training and lack of funder and institution expectations were identified as barriers to sharing data. Reviewers also raised concerns about lost publication opportunities, misuse of data by others, and criticisms when sharing review materials. Normalization and institutional factors are essential for promoting data‐sharing practices in evidence‐synthesis research.

## AUTHOR CONTRIBUTIONS

**Phi‐Yen Nguyen**: Data Curation, formal analysis, investigation, methodology, writing—original draft preparation. **Joanne E. McKenzie**: Conceptualization, methodology, supervision, writing—review and editing. **Daniel G. Hamilton**: Methodology, writing—review and editing. **Daniel G. Hamilton, Peter Tugwell, Fiona M. Fidler, Neal R. Haddaway, Julian P. T. Higgins, Raju Kanukula, Sathya Karunananthan, Lara J. Maxwell, Steve McDonald, Shinichi Nakagawa, David Nunan, Vivian A. Welch**: Writing—review and editing. **Matthew J. Page**: Conceptualization, funding acquisition, methodology, supervision, data curation, validation, writing—review and editing.

## CONFLICT OF INTEREST STATEMENT

The authors declare no conflict of interest.

## Supporting information

Supporting information.

Supporting information.

## Data Availability

This article has earned Open Data and Open Materials badges. Data and materials are available at https://osf.io/ch945/. This article has earned a Preregistered Research Designs badge for having a preregistered research design, available at https://research.monash.edu/en/publications/the-reprise-project-protocol-for-an-evaluation-of-reproducibility.
