# Supplementary material for: Systematic reviewers' perspectives on sharing review data, analytic code, and other materials: A survey
Source: Cochrane Evid Synth Methods. 2023 Apr 10;1(2):e12008. doi: 10.1002/cesm.12008 (PMC11795976; doi:10.1002/cesm.12008)
Supplement: Supplementary file 2 — Supporting information. [file CESM-1-e12008-s001.docx]

**Title page**

S1. Deviations from the REPRISE project protocol 2

S2. Invitation email template 4

S3. Survey form 5

S4. Classifications and illustrative quotes for additional materials respondents shared with their most recent systematic review 13

S5. Classifications and illustrative quotes for additional reasons respondents indicated for sharing their data file(s) or analytic code publicly alongside their most recent systematic review 14

S6. Classifications and illustrative quotes for additional reasons respondents indicated for not sharing their data file(s) or analytic code publicly alongside their most recent systematic review 15

S7. Views on sharing of systematic review data and analytic code 16

S8. Classifications and illustrative quotes for additional materials respondents suggested systematic reviewers should share publicly alongside their systematic review 20

**Supplementary File S1: Deviations from the REPRISE project protocol**

| **Original plan** | **Method implemented** | **Reason for modification** |
| --- | --- | --- |
| We would survey all systematic reviewers concurrently on (i) their views on sharing review data, analytic code and other materials and (ii) their understanding of and opinions about replication of systematic reviews. | We drafted two separate surveys addressing each of these topics and created separate equal samples of systematic reviewers for each of these surveys. | We anticipated this would help minimise respondent fatigue and improve survey completion rate. |
| We would derive the sampling frame for this study from a larger sampling frame, originally created for Study 1 of the REPRISE project. Study 1 involved systematically searching for systematic reviews of interventions indexed during a four-week period in late 2020 and evaluating their reporting characteristics. After running the searches, which yielded 6,292 unique records, we randomly sorted the records and screened 2,000 of them to meet our target sample of 300 systematic reviews. After excluding those 2,000 studies screened for REPRISE Study 1, the remaining 4,292 records constituted the sampling frame for Study 2. | We drew a new, larger sample of authors to survey using a new search strategy in PubMed: (meta-analysis[PT] OR meta-analysis[TI] OR systematic[sb]) AND 2021/01/01:2021/04/30 [EDAT] | In Study 1, 15% of the 2,000 records screened were eligible. Therefore, out of the remaining 4,292 records, we anticipated that only 640 records (15%) would meet the inclusion criteria. This left us with 320 authors to invite to complete each of the two surveys, which we considered too small a sample. |
| We would screen all remaining records using the inclusion criteria for REPRISE Study 1 and invite the corresponding authors of any eligible record (excluding those already included in Study 1). | We included all records retrieved by the new search in the sampling frame, regardless of whether they met the inclusion criteria used in REPRISE Study 1. We screened all titles to see whether the record was a correction, corrigendum, erratum, author’s reply or response related to a systematic review, and only included the systematic review in the sampling frame. | The new sampling frame was large (approximately 9,300 records), making it time-consuming to assess every record against the inclusion criteria in REPRISE Study 1. |
| We would send up to 3 reminders separated by 3 weeks. | We sent up to 3 reminders separated by 3 weeks in the pilot survey, and reduced the interval to 2 weeks in the main survey. | We needed to shorten the duration of the survey to avoid the holiday period (December to January). The pilot survey showed that most authors would have responded within the first 2 weeks. |

**Supplementary File S2. Invitation email template**

Hello,

We would like to invite you to participate in a project aiming to explore researchers' perspectives on data sharing for systematic reviews. Participation involves completing a survey, which will ask about your previous experiences with, and your views on, the sharing of systematic review data, analytic code and other materials. The survey is designed to capture the breadth of possible perspectives on this issue, and we are keen to hear from researchers such as yourself so that our findings best represent the views of those who conduct systematic reviews.

The survey takes approximately 15 minutes to complete. We anticipate that the knowledge gained will inform future guidance on data sharing and ultimately lead to higher quality systematic reviews.

If you would like to participate, follow this link to the Survey: [link]

Or copy and paste the URL below into your internet browser: [link]

**Consent:** Providing electronic consent will be accepted as a proxy for signing a consent form, a copy of which can be downloaded here or from the first page of the survey.

You can save your responses, then close the browser and return to the survey using the same link up to one week later. The survey is compatible with mobile devices.

**Where can I get further information?**

If you would like to discuss the study, please do not hesitate to contact Matthew Page (matthew.page@monash.edu). A participant information sheet providing further information about the study can be downloaded here.

We thank you for your time and expertise.

Dr Matthew Page

Project lead and Senior Research Fellow, Monash University, Australia

On behalf of the REPRISE investigator team: Joanne McKenzie, David Moher, Fiona Fidler, Julian Higgins, Neal Haddaway, Daniel Hamilton, Raju Kanukula, Sathya Karunananthan, Lara Maxwell, Steve McDonald, Shinichi Nakagawa, Phoebe Nguyen, David Nunan, Peter Tugwell, Vivian Welch

Follow the link to opt out of future emails: [link]

**Supplementary File S3. Survey Form**

**Part A: Views on replication of systematic reviews**

| **Item** | **Response options** |
| --- | --- |
| How often do you think systematic reviewers should share publicly each of the following items alongside their systematic review article (e.g. in an open-access data repository or supplementary file)? | |
| Full line-by-line search strategy as run in all databases | - Never - Rarely - Sometimes - Often - Always |
| File(s) containing citations of all records that were screened | - Never - Rarely - Sometimes - Often - Always |
| Template data collection form(s) | - Never - Rarely - Sometimes - Often - Always |
| File(s) containing (unprocessed) data extracted from included studies | - Never - Rarely - Sometimes - Often - Always |
| File(s) containing conversions to prepare data for analysis (e.g. code files to convert standard errors of the mean to standard deviations, or spreadsheets containing formulas) | - Never - Rarely - Sometimes - Often - Always |
| File(s) containing data used in all meta-analyses, subgroup analyses, sensitivity analyses and other analyses (e.g. spreadsheet, CSV file, or RevMan file containing effect estimates for each study) | - Never - Rarely - Sometimes - Often - Always |
| Analytic code used to generate results (e.g. text file or RMarkdown (.Rmd) file containing the sequence of commands used within a software package to analyse data) | - Never - Rarely - Sometimes - Often - Always |
| Metadata which describes the contents of the shared file(s) to aid interpretation and reuse (e.g. a file with complete descriptions of variable names (sometimes called a data dictionary), or README files describing each file shared) | - Never - Rarely - Sometimes - Often - Always |
| Other materials (please specify): _______________ | - Never - Rarely - Sometimes - Often - Always |
| **Considering the most recently published systematic review for which you were the corresponding author…** | |
| Which of the following items did you share publicly alongside your most recently published systematic review article (e.g. in an open-access data repository or supplementary file)? [Select all that apply] | 🞏 A. Full line-by-line search strategy as run in all databases  🞏 B. File(s) containing citations of all records that were screened  🞏 C. Template data collection form(s)  🞏 D. File(s) containing (unprocessed) data extracted from included studies  🞏 E. File(s) containing conversions to prepare data for analysis (e.g. code files to convert standard errors of the mean to standard deviations, or spreadsheets containing formulas)  🞏 F. File(s) containing data used in all meta-analyses, subgroup analyses, sensitivity analyses and other analyses (e.g. spreadsheet, CSV file, or RevMan file containing effect estimates for each study)  🞏 G. Analytic code used to generate results (e.g. text file or .Rmd file containing the sequence of commands used within a software package to analyse data)  🞏 H. Metadata which describes the contents of the shared file(s) to aid interpretation and reuse (e.g. a file with complete descriptions of variable names (sometimes called a data dictionary), or README files describing each file shared)  🞏 I. Other materials (please specify): _________ |
| *If D, E, F or G were selected:* Why did you share your data file(s) or analytic code publicly? [Select all that apply] | - The journal required sharing of data files or analytic code - The funder required sharing of data files or analytic code - My institution requires sharing of data files or analytic code - To enhance the transparency of my systematic review - To enhance the reproducibility of my systematic review - To gain more citations for my systematic review - To increase my chances of success in future job applications - To conform to expectations within my discipline - Other (please specify) |
| *If D, E, F or G were selected:* How were the data file(s) or analytic code publicly? [Select all that apply] | - Uploaded as a supplementary file on the journal website or as an appendix within the manuscript - Uploaded to a general-purpose open-access repository (e.g. Open Science Framework, Zenodo, GitHub, figshare) (please specify) - Uploaded to an institutional repository - Uploaded to my personal website - Other (please specify) |
| *If D, E, F or G were selected:* Are the shared file(s) associated with a persistent identifier (e.g. a Digital Object Identifier [DOI])? | - Yes, all files are associated with a persistent identifier - Yes, some files are associated with a persistent identifier - No - I do not know |
| *If D, E, F or G were selected:* Was a license applied to the file(s) shared (e.g. a Creative Commons license such as CC BY, CC BY-NC)? | - Yes, a license was applied to all files - Yes, a license was applied to some files - No - I do not know |
| *If F was NOT selected:* Why did you choose not to share the file(s) containing data used in all meta-analyses, subgroup analyses, sensitivity analyses and other analyses? [Select all that apply] | - I did not conduct any meta-analyses, subgroup analyses, sensitivity analyses or other analyses - I planned to conduct additional analyses of the data collected - I was concerned about misuse of the data file(s) - I did not see any value in sharing my data file(s) - I did not know how or where to share my data file(s) - I was too busy to prepare the data file(s) such that they could be used by someone external to the project - I wanted to protect my intellectual property - I wanted to protect commercially sensitive information - I was contractually obliged to not share the data file(s) - My organization has no one to advise on how to share data file(s) - My organization does not reward me for sharing data files (e.g. that is not captured in promotion criteria) - The journal did not request sharing of any data file(s) - I was concerned that others would criticize the data file(s) - I never considered sharing my data file(s) - Other (please specify) |
| Has anyone privately requested the data files, analytic code or other materials associated with your most recently published systematic review? | - Yes - No - I cannot remember |
| Did you register your most recently published systematic review (e.g. in PROSPERO, or the Open Science Framework) | - Yes - No - I cannot remember |
| **The following questions relate to possible factors that encourage or discourage sharing systematic review data file(s), that is, the file(s) containing data used in all analyses.**  **How much do you agree with the following statements?** | |
| I do not have permission to make any of my systematic review data file(s) publicly accessible. | - Strongly disagree - Disagree - Somewhat disagree - Neutral - Somewhat agree - Agree - Strongly agree |
| I would routinely share my systematic review data file(s) publicly if it were a common practice in my field/discipline. | - Strongly disagree - Disagree - Somewhat disagree - Neutral - Somewhat agree - Agree - Strongly agree |
| Preparing systematic review data file(s) to make them suitable for sharing is too time consuming for me. | - Strongly disagree - Disagree - Somewhat disagree - Neutral - Somewhat agree - Agree - Strongly agree |
| My funder expects me to share data file(s) publicly for all research projects, including systematic reviews. | - Strongly disagree - Disagree - Somewhat disagree - Neutral - Somewhat agree - Agree - Strongly agree |
| I prefer to share my systematic review data file(s) upon request because I can keep track of who has access to the files. | - Strongly disagree - Disagree - Somewhat disagree - Neutral - Somewhat agree - Agree - Strongly agree |
| I have received training in how to share my systematic review data file(s) publicly. | - Strongly disagree - Disagree - Somewhat disagree - Neutral - Somewhat agree - Agree - Strongly agree |
| Systematic review data file(s) are not important enough to share publicly. | - Strongly disagree - Disagree - Somewhat disagree - Neutral - Somewhat agree - Agree - Strongly agree |
| My institution expects me to share data file(s) publicly for all research projects, including systematic reviews. | - Strongly disagree - Disagree - Somewhat disagree - Neutral - Somewhat agree - Agree - Strongly agree |
| There is no suitable place to share my systematic review data file(s). | - Strongly disagree - Disagree - Somewhat disagree - Neutral - Somewhat agree - Agree - Strongly agree |
| If there are any other factors that encourage you to share your systematic review data file(s) that you would like to express, please record them here: | Text |
| If there are any other factors that discourage you from sharing your systematic review data file(s) that you would like to express, please record them here: | Text |
| **The following questions relate to possible consequences of sharing systematic review data file(s) and analytic code.**  **How much do you agree with the following statements?** | |
| I am concerned that if I shared my systematic review data file(s), other researchers might publish results based on the data before I am able to. | - Strongly disagree - Disagree - Somewhat disagree - Neutral - Somewhat agree - Agree - Strongly agree |
| I am concerned that if I shared my systematic review data file(s), other researchers might use the data for unintended purposes. | - Strongly disagree - Disagree - Somewhat disagree - Neutral - Somewhat agree - Agree - Strongly agree |
| I am concerned that if I shared my systematic review data file(s), alternative analyses will be performed, and it might be argued that my conclusions are not valid. | - Strongly disagree - Disagree - Somewhat disagree - Neutral - Somewhat agree - Agree - Strongly agree |
| I am concerned that if I shared my systematic review data file(s), readers might cite the paper(s) that have used my data more than they cite my original systematic review. | - Strongly disagree - Disagree - Somewhat disagree - Neutral - Somewhat agree - Agree - Strongly agree |
| I am concerned that if I share my systematic review data file(s) or analytic code, I will lose control over my intellectual property. | - Strongly disagree - Disagree - Somewhat disagree - Neutral - Somewhat agree - Agree - Strongly agree |
| I am concerned that if I share my systematic review data file(s) or analytic code, errors might be discovered in them, resulting in my results being deemed incorrect. | - Strongly disagree - Disagree - Somewhat disagree - Neutral - Somewhat agree - Agree - Strongly agree |
| I am concerned that if I share my analytic code, other researchers who examine the code might criticize me for conducting an incorrect analysis. | - Strongly disagree - Disagree - Somewhat disagree - Neutral - Somewhat agree - Agree - Strongly agree |
| If you have any other thoughts about sharing of systematic review data, analytic code and other materials which you would like to express, please record them here: | Text |

**Part B: General characteristics**

| **Item** | **Response options** |
| --- | --- |
| What is your primary country of residence? | Drop-down list of countries |
| What type(s) of institution are you affiliated with? [Select all that apply] | - University - Hospital - Government department - Commercial company - Research institute (not part of a university, hospital, government department or commercial company) - Other (please specify) |
| What research discipline do you primarily work in (e.g. medicine, nursing, psychology, economics, environmental sciences)? | Text |
| Do you conduct methodological research on systematic reviews? | - Yes - No |
| Are you a statistician? | - Yes - No |
| Are you a PhD student? | - Yes - No |
| Did you complete your PhD within the last 5 years? | - Yes - No - Not applicable (I do not have a PhD) |
| Approximately how many years have you been doing research? | - Less than 3 years - 3 to 10 years - More than 10 years |
| Approximately how many completed systematic reviews are you a co-author of (please count published and unpublished reviews)? | - None - One - Two - Three to 10 - More than 10 |
| In general, how do you feel about open science?  By open science, we mean the movement to make scientific content (e.g. publications, data, analytic code and other research materials) publicly available with minimal barriers to access (e.g. content is able to be viewed or downloaded without payment, registration or approval). | - Completely opposed - Mostly opposed - Slightly opposed - No opinion - Slightly support - Mostly support - Completely support |

**Supplementary File S4. Classifications and illustrative quotes for additional materials respondents shared with their most recent systematic review**

| **Classification** | **Illustrative quote** | **Freq. (%)** |
| --- | --- | --- |
| Citations of excluded full texts screened | “Information about all articles which we screened at full text review and main reason for rejection” | 8/417 (2%) |
| Risk-of-bias assessments | “File containing detailed quality and risk of bias assessments” | 8/417 (2%) |
| Protocol or registration record | “PROSPERO file” | 7/417 (2%) |
| Reporting checklist | “PRISMA checklist” | 4/417 (1%) |
| Details of screening and/or data collection process | “Detailed steps and information regarding the article selection and data extraction processes” | 3/417 (1%) |
| Sample search strategy | “Full line-by-line search strategy for PubMed” | 3/417 (1%) |
| Additional results not presented in the main paper | “Tables and figures for additional results not available the manuscript” | 2/417 (0.5%) |
| Infographic/policy brief | “Infographic summarising the article in an accessible format for all readers” | 2/417 (0.5%) |
| Data on adverse events | “Detailed adverse events data extracted” | 1/417 (0.2%) |
| Description of meta-analytic models used | “Detailed description of the models in the Supplementary material (with proper citations)” | 1/417 (0.2%) |
| Coding process for qualitative data | “Equivalent of analytical code in qualitative data review” | 1/417 (0.2%) |

**Supplementary Table S5. Classifications and illustrative quotes for additional reasons respondents indicated for sharing their data file(s) or analytic code publicly alongside their most recent systematic review**

| **Classification** | **Illustrative quote** | **Freq. (%)** |
| --- | --- | --- |
| To demonstrate good practices | “To show to editors/reviewers that we used good practices” | 2/157 (1%) |
| To overcome journal limits | “To share all extracted data, that was unable to be included in the manuscript due to space limits” | 2/157 (1%) |
| To facilitate future updates | “To allow others to update the review later when more studies come out” | 1/157 (1%) |
| To increase chance of journal acceptance | “To increase my chance of acceptance” | 1/157 (1%) |

**Supplementary Table S6. Classifications and illustrative quotes for additional reasons respondents indicated for not sharing their data file(s) or analytic code publicly alongside their most recent systematic review**

| **Classification** | **Illustrative quote** | **Freq. (%)** |
| --- | --- | --- |
| Had to comply with confidentiality requirements | “Files contained sensitive, personal data” | 2/265 (1%) |
| Shared files would need a lot of explanations/ annotations | “I would be happy to share on request. The analysis files are complex and would require me to explain how they work, which is why it makes more sense to give access on a case-by-case basis rather than access for everyone” | 2/265 (1%) |
| Sharing might be cumbersome for readers | “Sharing all the information might be cumbersome for both the journal and readers” | 1/265 (0.4%) |
| I do not own the data | “I am not the data owner and the data owners do not want to share them” | 1/265 (0.4%) |
| Funder does not mention that data/code need to be shared | “The funding agency has specific and strict rules on what is included in appendices, and these are not included” | 1/265 (0.4%) |

**Supplementary File S7. Views on sharing of systematic review data and analytic code**

|  | **Response   n/N (%)** |
| --- | --- |
| **Frequency that the following file types should be shared routinely** | |
| **Full line-by-line search strategy in all databases** |  |
| Often or always | 343/409 (84%) |
| Rarely or never | 18/409 (4%) |
| Sometimes | 48/409 (12%) |
| **Data collection template** |  |
| Often or always | 234/405 (58%) |
| Rarely or never | 70/405 (17%) |
| Sometimes | 101/405 (25%) |
| **Data used in meta-analyses** |  |
| Often or always | 228/404 (56%) |
| Rarely or never | 87/404 (22%) |
| Sometimes | 89/404 (22%) |
| **Citations of all screened records** |  |
| Often or always | 203/407 (50%) |
| Rarely or never | 108/407 (27%) |
| Sometimes | 96/407 (24%) |
| **Metadata of shared file(s)** |  |
| Often or always | 189/400 (47%) |
| Rarely or never | 109/400 (27%) |
| Sometimes | 102/400 (26%) |
| **Analytic code** |  |
| Often or always | 173/403 (43%) |
| Rarely or never | 133/403 (33%) |
| Sometimes | 97/403 (24%) |
| **Data preparation/conversion** |  |
| Often or always | 153/406 (38%) |
| Rarely or never | 134/406 (33%) |
| Sometimes | 119/406 (29%) |
| **Unprocessed extracted data** |  |
| Often or always | 151/407 (37%) |
| Rarely or never | 144/407 (35%) |
| Sometimes | 112/407 (28%) |
| **Other materials** |  |
| Often or always | 47/125 (38%) |
| Rarely or never | 53/125 (42%) |
| Sometimes | 25/125 (20%) |
| **Factors that can influence decision to share systematic review data file(s) *** | |
| **I would routinely share my systematic review data file(s) publicly if it were a common practice in my field/discipline.** |  |
| Agree | 280/357 (78%) |
| Disagree | 42/357 (12%) |
| Neutral | 35/357 (10%) |
| **Preparing systematic review data file(s) to make them suitable for sharing is too time consuming for me.** |  |
| Agree | 223/358 (62%) |
| Disagree | 96/358 (27%) |
| Neutral | 39/358 (11%) |
| **I prefer to share my systematic review data file(s) upon request because I can keep track of who has access to the files.** |  |
| Agree | 192/358 (54%) |
| Disagree | 107/358 (30%) |
| Neutral | 59/358 (16%) |
| **There is no suitable place to share my systematic review data file(s).** |  |
| Agree | 109/356 (31%) |
| Disagree | 153/356 (43%) |
| Neutral | 94/356 (26%) |
| **My funder expects me to share data file(s) publicly for all research projects, including systematic reviews.** |  |
| Agree | 67/356 (19%) |
| Disagree | 158/356 (44%) |
| Neutral | 131/356 (37%) |
| **My institution expects me to share data file(s) publicly for all research projects, including systematic reviews.** |  |
| Agree | 59/355 (17%) |
| Disagree | 191/355 (54%) |
| Neutral | 105/355 (30%) |
| **I do not have permission to make any of my systematic review data file(s) publicly accessible.** |  |
| Agree | 47/357 (13%) |
| Disagree | 263/357 (74%) |
| Neutral | 47/357 (13%) |
| **I have received training in how to share my systematic review data file(s) publicly.** |  |
| Agree | 42/356 (12%) |
| Disagree | 283/356 (79%) |
| Neutral | 31/356 (9%) |
| **Systematic review data file(s) are not important enough to share publicly.** |  |
| Agree | 41/358 (11%) |
| Disagree | 271/358 (76%) |
| Neutral | 46/358 (13%) |
| **Consequences of sharing systematic review data file(s) and analytic code** | |
| **Other researchers might publish results using my data before me.** |  |
| Agree | 180/357 (50%) |
| Disagree | 143/357 (40%) |
| Neutral | 34/357 (10%) |
| **Other researchers might use data for unintended purposes.** |  |
| Agree | 171/356 (48%) |
| Disagree | 141/356 (40%) |
| Neutral | 44/356 (12%) |
| **I might lose control over my intellectual property.** |  |
| Agree | 141/355 (40%) |
| Disagree | 159/355 (45%) |
| Neutral | 55/355 (15%) |
| **Readers might cite the paper(s) that used my data more than my review.** |  |
| Agree | 118/355 (33%) |
| Disagree | 177/355 (50%) |
| Neutral | 60/355 (17%) |
| **My code might by criticized by others as an incorrect analysis.** |  |
| Agree | 112/352 (32%) |
| Disagree | 190/352 (54%) |
| Neutral | 50/352 (14%) |
| **Alternative analyses might be performed and used to argue against my conclusions.** |  |
| Agree | 112/356 (31%) |
| Disagree | 191/356 (54%) |
| Neutral | 53/356 (15%) |
| **Errors might be discovered in my data or code, resulting in my results being deemed incorrect.** |  |
| Agree | 101/354 (29%) |
| Disagree | 200/354 (56%) |
| Neutral | 53/354 (15%) |

* *Three levels of agreement were created by aggregating the 7-point Likert scale.*

*Agree = Somewhat agree or Agree or Strongly agree*

*Disagree = Somewhat disagree or Disagree or Strongly disagree*

*Neutral = Neutral*

**Supplementary File S8. Classifications and illustrative quotes for additional materials respondents suggested systematic reviewers should share publicly alongside their systematic review**

| **Classification** | **Illustrative quote** | **Freq. (%)** |
| --- | --- | --- |
| ***Frequency: Always*** |  |  |
| Protocol or registration record | “PROSPERO registration” | 7/125 (6%) |
| Details of screening/data collection processes | “Full screening and extraction descriptive methodology” | 5/125 (4%) |
| Risk-of-bias assessments | “Risk of bias assessment: template and ratings” | 4/125 (3%) |
| Citations of excluded full texts screened | “Excluded full-text articles” | 3/125 (2%) |
| Detailed outline of review workflow | “Any additional workflow or strategies” | 3/125 (2%) |
| Reporting checklist | “Reporting checklists” | 2/125 (2%) |
| Platform used to search databases | “Platforms used” | 1/125 (1%) |
| Data on inter-rater agreement for screening | “Information on the agreement between reviewers for the study selection process (Kappa and data for its calculation)” | 1/125 (1%) |
| Data on adverse events | “Detailed adverse events data extracted” | 1/125 (1%) |
| ***Frequency: Often*** |  |  |
| Risk-of-bias assessments | “Full RoB judgements and rationales” | 2/125 (2%) |
| Reasons for exclusion of studies | “Reasons of excluded studies” | 1/125 (1%) |
| Additional results not presented in the main paper | “Tables and figures for additional results not available the manuscript” | 1/125 (1%) |
| Search results for each database | “A file of generic link to search results for each database” | 1/125 (1%) |
| ***Frequency: Sometimes*** |  |  |
| Mathematical formulae used | “Documents explaining mathematical formulae and additional citations” | 1/125 (1%) |
| Risk-of-bias assessments | “Risk of bias raw data files” | 1/125 (1%) |
